# Gastric Cancer Cell Lines Have Different* MYC*-Regulated Expression Patterns but Share a Common Core of Altered Genes

**DOI:** 10.1155/2018/5804376

**Published:** 2018-10-16

**Authors:** Jersey Heitor da S. Maués, Helem Ferreira Ribeiro, Giovanny R. Pinto, Luana de Oliveira Lopes, Letícia M. Lamarão, Carla Mariana F. Pessoa, Caroline de Fátima Aquino Moreira-Nunes, Raimundo Miranda de Carvalho, Paulo P. Assumpção, Juan A. Rey, Rommel M. Rodríguez Burbano

**Affiliations:** ^1^Human Cytogenetics Laboratory, Institute of Biological Sciences, Federal University of Pará, Belém, Brazil; ^2^Center of Biological and Health Sciences, Department of Biomedicine, University of Amazon, Belém, Brazil; ^3^Department of Biomedicine, Federal University of Piauí, Parnaíba, Brazil; ^4^Laboratory of Nucleic Acids, State Center of Hematology and Hemotherapy, Belém, Brazil; ^5^Oncology Research Nucleus, University Hospital João de Barros Barreto, Federal University of Pará, Belém, Brazil; ^6^Laboratory of Pharmacogenetics, Drug Research and Development Center, Federal University of Ceará, Fortaleza, Brazil; ^7^Laboratory of Molecular Biology, Ophir Loyola Hospital, Belém, Brazil; ^8^Molecular Oncogenetics Laboratory, Research Unit, Hospital Universitario La Paz, Madrid, Spain

## Abstract

*MYC* is an oncogene responsible for excessive cell growth in cancer, enabling transcriptional activation of genes involved in cell cycle regulation, metabolism, and apoptosis, and is usually overexpressed in gastric cancer (GC). By using siRNA and Next-Generation Sequencing (NGS), we identified* MYC*-regulated differentially expressed Genes (DEGs) in three Brazilian gastric cancer cell lines representing the histological subtypes of GC (diffuse, intestinal, and metastasis). The DEGs were picked using* Sailfish* software, followed by Gene Set Enrichment Analysis (GSEA) and Kyoto Encyclopedia of Gene and Genome (KEGG) pathway analysis using KEGG. We found 11 significantly enriched gene sets by using enrichment score (ES), False Discovery Rate (FDR), and nominal P-values. We identified a total of 5.471 DEGs with correlation over (80%). In diffuse-type and in metastatic GC cell lines,* MYC*-silencing caused DEGs downregulation, while the intestinal-type GC cells presented overall DEGs upregulation after* MYC* siRNA depletion. We were able to detect 11 significant gene sets when comparing our samples to the hallmark collection of gene expression, enriched mostly for the following hallmarks: proliferation, pathway, signaling, metabolic, and DNA damage response. When we analyzed our DEGs considering KEGG metabolic pathways, we found 12 common branches covering a wide range of biological functions, and three of them were common to all three cell lines: ubiquitin-mediated proteolysis, ribosomes, and system and epithelial cell signaling in* Helicobacter pylori* infection. The GC cell lines used in this study share 14* MYC-*regulated genes, but their gene expression profile is different for each histological subtype of GC. Our results present a computational analysis of* MYC*-related signatures in GC, and we present evidence that GC cell lines representing distinct histological subtypes of this disease have different* MYC*-regulated expression profiles but share a common core of altered genes. This is an important step towards the understanding of* MYC*'s role in gastric carcinogenesis and an indication of probable new drug targets in stomach cancer.

## 1. Introduction

Gastric cancer (GC) remains as an important cause of cancer-related morbidity and mortality worldwide, with recent estimates accounting for over 950.000 new diagnosis and 720.000 deaths each year [1]. Treatment of GC at advanced stages remains difficult, and the prognosis is still poor, partly as a result of local recurrence, tumor invasion, and/or metastasis [2]

The* MYC *oncogene, located at 8q24, is a key oncogene in gastric carcinogenesis, and an increase in both copy number and mRNA expression was classified as one of the driver mutations in gastric tumors [3].* MYC* amplification and overexpression are present in 6-58% of all sporadic gastric tumors [4–6], being more frequent in Brazilian samples [7–9], usually as a result from gene amplification and chromosomal translocations [2, 10].

Our research group previously reported that MYC mRNA and protein overexpression is a common finding in GC samples and in some preneoplastic gastric lesions [7, 11–14] from a Brazilian population, as well as in nonhuman primate models of gastric carcinogenesis [15]. We also established and characterized three GC cell lines, AGP01, ACP02, and ACP03, obtained from intestinal-type GC metastasis, diffuse-type GC, and intestinal-type GC, respectively (Leal et al. 2009). Those cell lines also carry genetic alterations commonly found in Brazilian GC patients, such as* MYC* amplification and overexpression and* TP53 *deletion [7, 13, 16].

Some consequences of excessive intracellular* MYC* levels are genomic instability [17] and error-prone DNA replication caused by oncogene-induced replicative stress [18]. Even though there is an association between an increase in* MYC* expression and gastric cancer, its exact role in gastric tumorigenesis is not yet fully understood [19, 20] and most of the high-throughput studies carried so far concerning gastric cancer genetics overlook* MYC*'s importance in this process [2, 3, 21–24]

Bioinformatics has mostly been applied in basic science research. Following the completion of human genome sequencing, it has also facilitated numerous discoveries in basic medicine, and several clinical applications of bioinformatics have been reported, including clinical sequencing, an emerging field of precision medicine [25]. In cancer research, bioinformatics has been used to study cancer transcriptome, early diagnosis, cancer grading, and prognosis prediction [26].

In this study, we used RNA interference (RNAi) to block* MYC*'s mRNA translation, followed by Ion Proton™ semiconductor sequencing, in order to identify* MYC*'s regulation signature in AGP01, ACP02, and ACP03 cell lines. We found 11 common pathways for the GC cell lines, which we believe can help in the understanding of expression signatures in different GC histological subtypes.

## 2. Materials and Methods

### 2.1. Cell Lines and siRNA Transfection

Three GC cell lines previously established and characterized by our group were used: AGP01, ACP02, and ACP03 [27]. The three cell lines present chromosome 8 trisomy,* MYC* amplification [13, 27], and* TP53* deletion, which are common genetic alterations in Brazilian gastric cancer patients [28] and in another GC cell line developed in Brazil [29]. A cell culture of nonneoplastic gastric mucosa cells (MNP01, Normal Gastric Mucosa Cell Line 01) pooled from 10 patients without gastric cancer or any other gastric disease, was also used to evaluate the gene and protein expression after* MYC*-silencing and to validate the knockdown results, as well as the* MYC-*regulated genes identified after NGS.

A total of 3x10^5^ cells were seeded into 6 cm^2^ plates for each cell line for 24 h before transfection. Small interfering RNAs (siRNA) targeting* MYC* (ON-TARGETplus Human MYC (4609) siRNA Dharmacon, EUA) or scrambled control siRNAs (ON-TARGETplus Non-Targeting Pool, Dharmacon, EUA) were transfected into AGP01, ACP02, and ACP03 cell lines using Lipofectamine RNAiMAX Transfection Reagent (Thermo Fisher Scientific, EUA). Optimal transfection was reached after 48 h, and total RNA and proteins were extracted with TRIzol reagent (Thermo Fisher Scientific, EUA). All siRNA experiments were performed three times. The sample names and GEO access codes are shown in Table 1. All siRNA experiments were carried out in biological triplicates.

### 2.2. Semiconductor Sequencing and Data Pretreatment

Total RNA samples were first treated with DNAse-I to remove any possible DNA contamination, and then the mRNA was enriched using Dynabeads Oligo(dt)_25_ (Thermo Fisher Scientific, USA). The enriched mRNA was fragmented in smaller fragments of 200 bps approximately, which were attached to adapters with known sequences that were unique for each sample. Samples were connected to magnetic beads containing complementary sequences for the adapters and then inserted in microwells where an emulsion-PCR for cDNA synthesis was carried (illustra Ready-To-Go RT-PCR Beads, GE Lifesciences). Our six cDNA libraries were submitted to quantification and quality control using Agilent 2100 Bioanalyzer and were then loaded in Ion Proton V2 PI chip using the Ion PI™ 200 Sequencing Kit v3 and sequenced using Ion Proton™ (Thermo Fisher Scientific, EUA) platform in a single multiplex run.

Raw data reads obtained by primary sequencing using Ion Proton™ were submitted to quality control to calculate alignment and to assess how the reads behave when compared to the reference human genome (Hg19/GRCh37). The aligned reads were mapped and quantified using TMAP (*Torrent Mapping Alignment Program*), which supports different alignment algorithms [30–32]. Processed datasets were uploaded to GEO (Gene Expression Omnibus) under the access number GSE81265.

### 2.3. Identification and Statistical Analysis of DEGs

Sailfish software pack [33] and the RPKM (Reads Per Kilobase per million mapped reads) [34, 35] were used to significance analysis of DEGs between control and* MYC*-silenced samples. To identify the DEGs between two paired samples, we used the Audic-Claverie test [36]. Fold-change (FC) was calculated as the log_2_ ratio between the silenced (M) and the control (C) sample. We used a p-value correction corresponding to differential expression tests using Bonferroni correction [37]. Our cut-off for DEGs definition was established as a False Discovery Rate (FDR) < 0.05 [38] and |log2 (FC)| > 1. DEGs were plotted using Multiplot v2, which exhibits personalized gene expression profiles (http://software.broadinstitute.org/cancer/software/genepattern/modules/docs/multiplot/2).

### 2.4. Functional Enrichment Analysis

We used Gene Set Enrichment Analysis (GSEA) [39] to identify significantly enriched gene sets between siRNA control versus MYC-silenced and vice versa. The gene expression changes perceived by DEGs were related to biologically enriched pathways found in GSEA. The gene expression datasets used were collections H (Hallmark gene set) [40] and C2 (curated gene set: KEGG), publicly available at MsigDB [41]. The standard parameters defined by Subramanian et al. were used in our analysis. The statistical significance of GSEA analysis was determined by 100 permutations, the enrichment maps were created to significant (*P* < 0.05 and False Discovery Rate (FDR) < 0.25) gene sets, and GraphPad Prism™ Software was used to graphically represent our data.

### 2.5. Real-Time Quantitative PCR (RT-qPCR)

To confirm the silencing effect of siRNA on* MYC* expression, we used real-time quantitative PCR to evaluate its expression in relation to the expression found for the normal gastric mucosa cell line MN01. All tests were made in triplicate and using TaqMan® probes as assays-on-demand products for gene expression (Life Technologies, EUA) (*MYC: *Hs00153408_m1). The fourteen DEGs identified by our analysis (*SKIV2L2: *Hs00299011_m1*, SRPRB: *Hs00253639_m1*, JUNB: *Hs00357891_s1*, BNIP3: *Hs00969291_m1*, RAB22A: *Hs00221082_m1*, TMED2*: Hs00607277_m1*, ACAT1*: Hs00608002_m1*, NDUFV2*: Hs00221478_m1*, LBR*: Hs01032700_m1*, NCL*: Hs01066668_m1*, AAAS*: Hs00210351_m1*, ATXN2*: Hs00268077_m1*, LGMN*: Hs00271599_m1, and* CDKN1B*: Hs00153277_m1) were also analyzed and validated by real-time quantitative PCR. The expression of those genes was calculated relative to their expression in the normal gastric mucosa cell line MN01.

## 3. Results

### 3.1. Quantification of GCs Transcripts and Identification of DEGs Using NGS

In this study, we used next-generation sequencing based in semiconductors, as well as RNA-Seq, to quantify the transcripts and its isoforms in three gastric cancer cell lines, ACP02, ACP03, and AGP01, before and after* MYC*-silencing using siRNA. The use of siRNA to reduce* MYC *expression in the three gastric cancer cell line used in this study was very effective, reducing* MYC* mRNA expression in 73% for AGP01, in 84% for ACP02, and in 77% for ACP03.

Our NGS sequencing of six libraries generated over 75 million reads, which, after enrichment, were mapped within the reference genome in over 99% of the samples and in over 98% of the reference transcriptome (Table 2), and the distribution of the amplified segments was consistent in all samples. Table 2 also shows in average how many genes were identified for each sample. The average reads produced by Ion Proton™ ranged between 125 and 130 bps.

**Figure 1:**
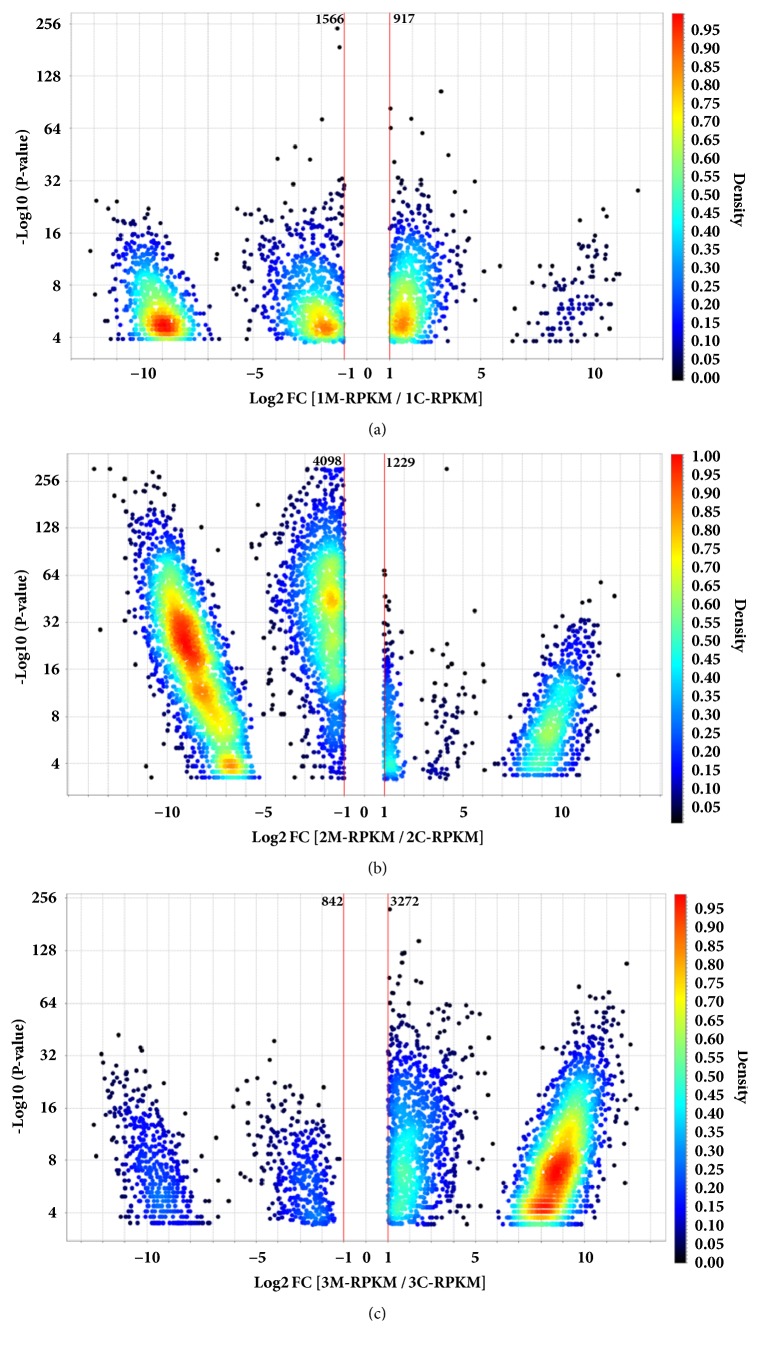
Volcano plots of DEGs for three GC cell lines after* MYC*-silencing. log_2_ Fold-change and P-values (-log_10_) are shown for DEGs with (|log_2_ (FC)| > 1 and p ≤ 0.05). (a) DEGs for AGP01. (b) DEGs for ACP02. (c) DEGs for ACP03. Density is a special-case calculation which is only used in advanced shading. It considers only the operands for this calculation are the X and Y values of the graph axes.

According to our cut-off (FDR < 0.05 and |log_2_ (FC)| > 1), we obtained a distinct amount of DEGs between siRNA control versus* MYC* -silenced samples. Using Multiplot (v2), we identified 1.556 downregulated and 917 upregulated DEGs for AGP01; for the diffuse-type cell line (ACP02), we found 4.098 downregulated versus 1.229 upregulated DEGs; finally, for ACP03, an intestinal-type cell line, we identified 3.272 upregulated versus 842 downregulated DEGs (Figure 1). Our results indicate that it is possible to discern histological subtypes of GC by analyzing its* MYC*-related gene expression pattern.

**Figure 2:**
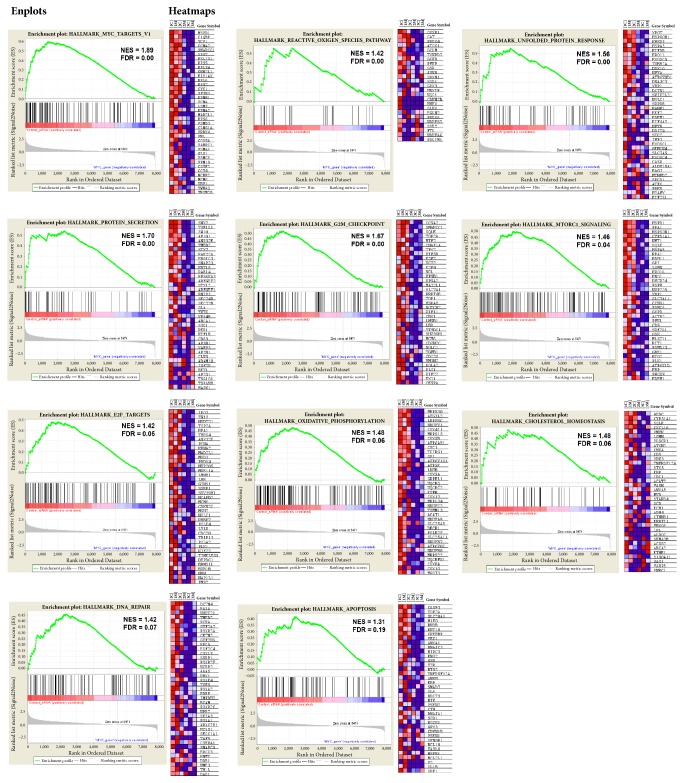
Panel with 11 gene sets enriched for three GC cell lines after* MYC*-silencing. Each gene set was represented on the Enplot by its normalized enrichment score (NES) and False Discovery Rate (FDR), along with its heatmap showing the gene expression for each gene.

A total of 16.777 genes from our six datasets obtained by RNA-Seq (GSE81265) were inserted in an expression matrix normalized by RPKM and, after that, used for enrichment comparing with expression datasets from collections H and C2. By applying GSEA, we looked for gene sets which presented enrichment only in siRNA control samples, but not in* MYC* -silenced samples, likely* MYC* targets, and found 11 significant gene sets (*P* < 0.05 and FDR < 0.25), as shown in Table 3. We found a total of 7903 genes, and 5471 (69.2%) are enriched in siRNA control samples, presenting a very high correlation between biological replicates and libraries (80%). The enrichment maps obtained are shown in Figure 2, where we created a panel of 11 gene sets significantly enriched.

### 3.2. *MYC*-Silenced GC Cells Lines from Different Histological Subtypes Show Distinct* MYC*-Dependent Expression Profiles

**Figure 3:**
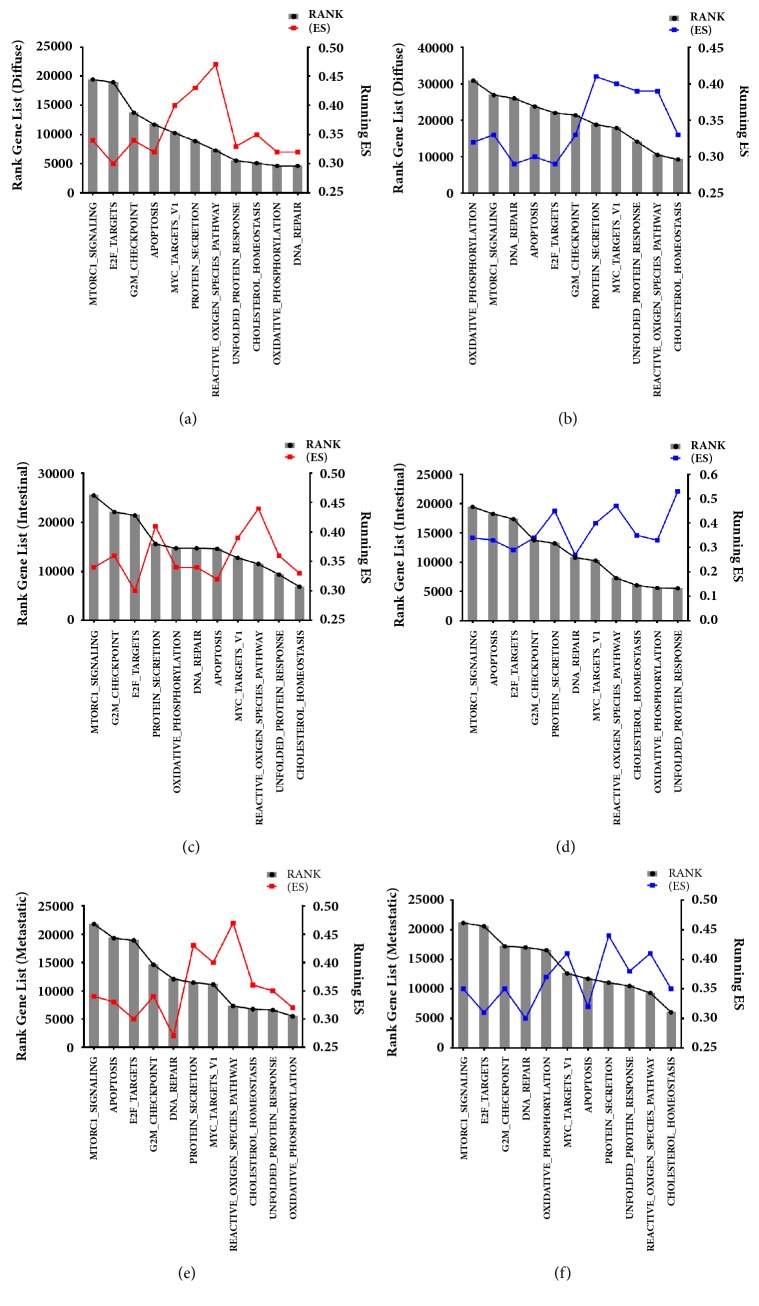
Ranks for enriched DEGs when compared to collection H with their average enrichment score (ES) for each analysis. (a) ACP02 upregulated DEGs, ranks (4.640 to 19.433), and ES (0.30 to 0.47). (b) ACP02 downregulated DEGs, ranks (9.245 to 30.875), and ES (0.29 to 0.41). (c) ACP03 upregulated DEGs, ranks (6.853 to 25.475), and ES (0.30 to 0.47). (d) ACP03 downregulated DEGs, ranks (5.530 to 19.433), and ES (0.27 to 0.53). (e) AGP01 upregulated DEGs, ranks (5.480 to 21.804), and ES (0.27 a 0.47). (f) AGP01 downregulated DEGs, ranks (6.060 to 21.134), and ES (0.30 a 0.44).

We then evaluated the metabolic pathways more likely to be* MYC*-regulated as shown by GSEA, listing those genes in the categories described as hallmarks of gene expression [40] and found that they are related to cellular proliferation, pathway, cellular signaling, metabolic processes, and response to DNA damage (Table 3). We refined the raw data sets obtained from our GC cell lines, identifying the DEGs for each of the 11 gene sets for collection H that were enriched for the Control_siRNA phenotype.  Figure 3 shows the ranks for enriched DEGs when compared to collection H with their average enrichment score (ES) for each analysis. Part of these results generated in this analysis is in Table 1 (see Supplementary Materials), which show the most enriched DEGs in the set gene MYC TARGETS V1.

The diffuse-type cell line (ACP02) presented 149 enriched DEGs by GSEA, 3 up- and 146 downregulated. Genes enriched with ranks above 15.000 fit the downregulated profile and were emphasized for gene sets such as* MYC* target V1, protein secretion, and reactive oxygen species pathway (Figure 3(a)). On the other hand, ranks above 30.000 were enriched for downregulated DEGs, presenting gene sets described as protein secretion,* MYC* target V1, unfolded protein response, and reactive oxygen species pathway (Figure 3(b)). Meanwhile, the intestinal-type cells (ACP03) showed 85 DEGs, 76 up- and 9 downregulated; genes with ranks above 20.000 were upregulated for pathways such as protein secretion,* MYC* target V1, and reactive oxygen species pathway (Figure 3(c)); downregulated genes with ranks above 15.000 presented enrichment emphasis for GM2-checkpoint, protein secretion,* MYC* target V1, reactive oxygen species pathway, and unfolded protein response (Figure 3(d)). For the metastatic samples (AGP01), 65 enriched DEGs were identified by GSEA, 25 up- and 40 downregulated, and the gene sets were enriched with ranks above 20.000 for both DEGs profiles. Upregulated sets (Figure 3(e)) were enriched for GM2-checkpoint, protein secretion,* MYC* target V1, and reactive oxygen species pathway, while the downregulated profile had emphasis for Oxidative_Phosphorylation,* MYC* target V1, protein secretion, and reactive oxygen species pathway (Figure 3(f)).

**Figure 4:**
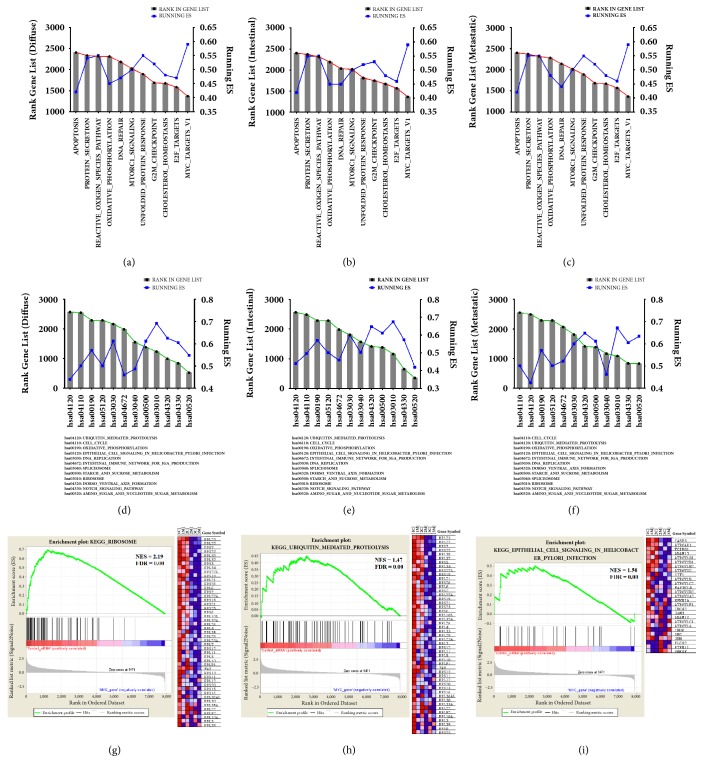
Identification of individual genes in the enriched DEG datasets with the higher ES. (a) DEGs enrichment for ACP02. (b) DEGs enrichment for ACP03. (c) DEGs enrichment for AGP01. The most enriched hallmark for the three cell lines as MYC_Target_V1 with an ES = 0.59. (d) KEGG pathway identification for ACP02. (e) KEGG pathway identification for ACP03. (f) KEGG pathway identification for AGP01. Enplot and heatmap for the pathways with the highest ES scores among the three GC cell lines can be seen in (g) for ACP02, in (h) for ACP03, and in (i) for AGP01.

In the 11 gene sets from collection H that were used for DEGs enrichment, we identified the genes with higher ES. We used this strategy to identify similar genes that overlap as a consensus grouping as described elsewhere [40], so it would be easier to identify* MYC*-regulated genes. We identified DEGs for the three GC cell lines that were enriched when compared to collection H, with ranks above 2.000. The MYC target V1 gene set was identified as the most enriched for our studied cell lines, with higher ES scores for the genes* SNRPD2* and* TYMS*. When we compared the enriched genes found for collection H for ACP02 and ACP03, we noticed that some genes presented enrichment ranks that were also common for the metastatic cells (AGP01) (Figures 4(a)–4(c)).

The same analysis was applied to identify enriched DEGs found for KEGG pathways, to deepen our understanding about the genes involved in the* MYC*-related carcinogenesis for the stomach. The enrichment maps for KEGG [42] are shown in Figures 4(d)–4(f), presenting 12 significantly enriched pathways found using GSEA, suggesting that GC has multiple altered pathways that lead normal gastric mucosa into the carcinogenic process. These pathways were defined by a normalized enrichment score (NES) > 1.47. The average t-statistic for the genes was calculated for each KEGG pathway using permutation tests with 100 repetitions. The enrichment plot for three common pathways altered in all our cell lines is presented in Figure 4(g) (ribosome-hsa03010), which presented the highest ES among the cells, Figure 4(h) (ubiquitin-mediated proteolysis-hsa04120), with the highest ranks in our gene list, and Figure 4(i) (epithelial cell signaling in* Helicobacter pylori* infection-hsa05120). For more details on the DEGs enriched with KEGG, see Table 2 in Supplementary Materials.

**Figure 5:**
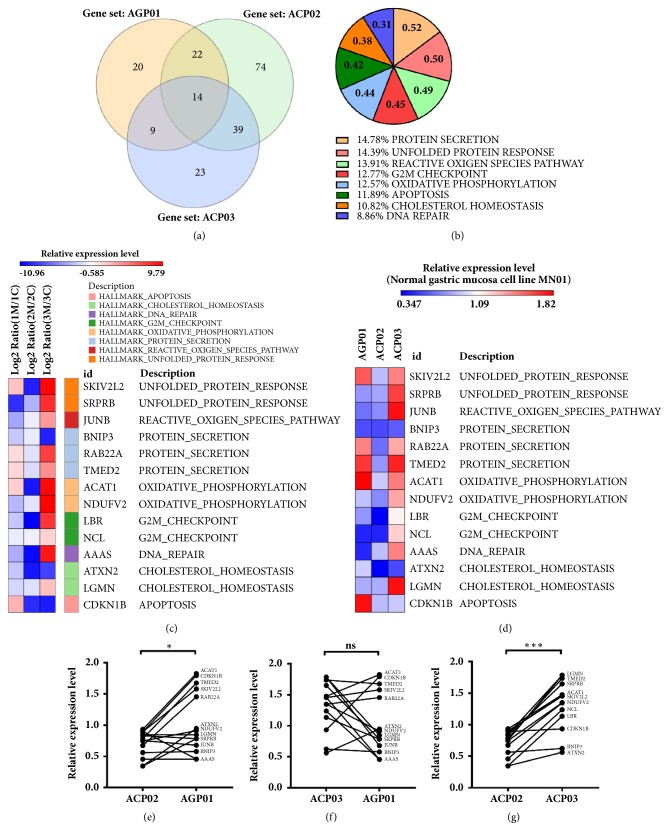
Common DEGs enriched in three GC cell lines after* MYC*-silencing. (a) Using Venn diagram, we were able to identify 14 common DEGs among AGP01, ACP02, and ACP03, but each one of them also has a unique set of DEGs. (b) Identification of the 14 common DEGs in 8 enriched hallmarks of collection H, represented by the % of its correspondent ES, ES scores are shown inside the pie chart for each hallmark. (c). Heatmap for the expression of the 14 common DEGs shared for three GC cell lines after* MYC*-silencing. Blue represents downregulation while red means upregulation. Notice that, even for the 14 common genes, each cell line has a different expression pattern. Relative read expression was normalized using log_2_ Fold-change between* MYC-*silenced/control siRNA. (d) Heatmap for the relative gene expression obtained by RT-qPCR for the 14 identified DEGS for the AGP01, ACP02, and ACP03 cell lines. (e) The overall expression for the 14 DEGs is downregulated in the ACP02 cell line when compared to AGP01 cells. (f) The gene expression levels were increased in ACP03 cell line, but no statistical difference was found. (g) Relative gene expression was increased for ACP03 when compared to ACP02. Wilcoxon matched-pairs signed rank test was used to compare the relative gene expression levels (^∗^P <0.05, ns: not significant, and ^∗∗∗^P <0.0001).

We identified enriched DEGs common to all three GC cell lines used in this study and represent them as Venn diagrams constructed by the InteractiVenn platform [43]. We found 14 DEGs that are shared enriched in both AGP01, ACP02, and ACP03 cell lines (Figure 5(a)), which are likely to be* MYC* targets according to our analysis. When we search for gene function in different databases (Figure 5(b)), most of them (14.78%) are involved in protein secretion, followed by unfolded protein response (14.39%) and reactive oxygen species pathway (13.91%).  Table 4 shows the 14 individual DEGs as well as the gene expression hallmarks they are involved, ranks and ES. We show a heatmap (Figure 5(c)) of the 14 shared DEGs in clusters grouped using the GENE E tool (https://software.broadinstitute.org/GENE-E/), where downregulation is expressed in blue and upregulation in red. Most genes for ACP02 (diffuse-type GC) presented downregulation, while for ACP03 (intestinal-type GC) the same genes presented themselves as upregulated. The metastatic cell line AGP01, even though its original tumor was intestinal-type, presented mixed expression patterns with predominant downregulation.

### 3.3. The 14* MYC-*Regulated DEGs Show Distinct Expression Profiles for Each GC Histological Subtype

Our* in silico* analysis identified a core set of 14 DEGs (Figure 5(a)) who are* MYC-*regulated, but whose expression profile is distinct for each GC cell line since they represent different histological subtypes. We validated the gene expression profiles presented by the AGP01, ACP02, and ACP03 after* MYC-*silencing by RT-qPCR in the cDNA obtained originally for each cell line and compared them with their expression in the MN01 cell line. Our results point out that the 14 identified DEGs are under* MYC* transcriptional regulation (Figure 5(d)). The ACP02 cell line presented mostly downregulation for the expression of the 14 DEGs (Figure 5(c)); on the other hand, ACP03, the same 14 genes, were upregulated; AGP01 results were mixed, with some genes showing downregulation and others showing downregulation. Our gene expression results (Figure 5(d)) confirm our* in silico* analysis (Figure 5(c)).

Each cell line used in this study represents a GC histological subtype: AGP01 was obtained from the ascitic fluid of intestinal-type GC, representing a metastatic disease, while ACP02 was developed from a diffuse-type stomach cancer patient and ACP03 origin was an intestinal-type gastric tumor.

We compared the mRNA expression measured by RT-qPCR for our identified DEGs to assess whether the gene expression of those 14 genes was enough to statistically distinguish each cell line. When comparing ACP02 versus AGP01 (Figure 5(e)), we noticed a significant gene expression downregulation for ACP02; confronting ACP03 versus AGP01 indicated an increase in mRNA relative quantification (Figure 5(f)) for ACP03; however, those results were not significant; the comparison between ACP02 versus ACP03 confirmed that the 14* MYC-*regulated DEGs are significantly more expressed in the ACP03, the intestinal-type cell line, than in ACP02 (Figure 5(g)).

Taken together, our results indicate that, even though* MYC*-related carcinogenesis alters the same 14 genes in GC cell lines representing the most common histological subtypes, how* MYC* causes GC carcinogenesis is different for each disease presentation and it is possible to distinct them by using expression signatures.

## 4. Discussion

A key goal of cancer studies is to systematically characterize the cellular and molecular mechanisms involved in the disease and its distinct stages, to identify both potential biomarkers and new probable drug targets [44]. The molecular profile of gastric cancer is heterogeneous, partly due to different classification systems [45] and, in order to clarify the true molecular origins of GC, both the Cancer Genome Atlas [22] and the Asian Cancer Research Group [46] published the molecular subtypes of gastric cancer, with remarkable overlap between the two models. Therefore, several genes have been implied as biomarkers for GC subtypes, such as* RHOA, EGFR, PDL, CDH1, TP53, *and* JAK2*. However, those studies use samples from populations in which the disease incidence is highest, and few studies have examined populations in which the incidence of this disease is lower, such as Brazil [47]. There is evidence that GC incidence varies between countries greatly because the genetic heterogeneity exhibited by human populations [48], and it has already been showed that there is a unique gene expression signature for Brazilian cases of intestinal-type GC [47]. Our study helps to highlight the molecular profiles of Brazilian GC cell lines, which can help greatly our understanding about the molecular basis of GC in South America.

Most NGS studies investigate GC by comparing tumor versus nontumoral tissue, analyzing global gene expression patterns, copy number variation, and other molecular characteristics, and most of the high-throughput studies carried so far concerning gastric cancer genetics overlook* MYC*'s importance in this process [2, 3, 21–24]. Our results are relevant because* MYC* overexpression is a key finding in Brazilian GC samples [8]. Therefore, we reduced the expression of this gene using siRNA to identify the* MYC-*related signature in GC cell lines, comparing nonsilenced with silenced samples. We identified a total of 5.471 DEGs, and 11 significant gene sets, including classic* MYC* targets represented by* MYC* target V1.

We hereby present the computational analysis of gene sets identified after* MYC*-silencing in Brazilian GC cell lines [13, 27], who carry genetic alterations commonly found in Brazilian GC patients [7, 13, 16]. This oncogene promotes cell growth acting as a transcription factor regulating cell cycle, metabolism, and cell survival [49]. We found DEGs upregulation only for the intestinal-type cell line (ACP03), while the diffuse-type (ACP02) and the metastatic GC cells (AGP01) presented overall gene expression downregulation; when looking at individual genes between ACP02 and AGP01, it is still possible to distinguish between them by* MYC-*related gene expression. It is important to notice that* MYC* has a dual-role in the carcinogenic process, selectively activating and inactivating different gene sets [50–52]. Taken together, we present evidences supporting the fact that* MYC* deregulation has an important role in gastric carcinogenesis [14] and that* MYC*-related signatures in gastric cancer are different for each histological subtype of this disease, which is clinically relevant [47, 53].

One of the main forms of MYC protein regulation in normal cells is through its targeted degradation by the ubiquitin-proteasome system [54], which was one of the KEGG enriched pathways found in our analysis (Figure 4). This means that, in a* MYC*-overexpression condition, like GC [7], not only this gene and its protein are more produced, but they are also less destroyed because it diminishes the expression of E3 ubiquitin-ligases, such as Fbw7 and HectH9, contributing to prolonged MYC protein half-life and amplification of its effects [55]. Ubiquitin-ligases, including the MYC-regulated Fbw7, have recently evolved as promising therapeutic targets for the development of novel anticancer drugs [56].

Other pathways involved in MYC-related gastric carcinogenesis found by our study are known targets, such as ribosome and cell cycle control genes, which are hardly druggable. When taken together, the 12 different pathways we found under* MYC* control for gastric carcinogenesis represent many biological functions, meaning that* MYC* overexpression in GC disturbs almost all the regular cellular processes in favor of tumor development [52]. Another interesting gene set includes glucose metabolism, which is an area of growing interest in cancer research [57], and it has been shown that* MYC* directs the activation of aerobic glycolysis, a hallmark of cancer metabolism known as Warburg effect, and pretty much all genes involved in glycolysis and most of the ones responsible for glutaminolysis [57, 58].

We were also able to pinpoint 14 enriched DEGs in all the three GC cell lines used in this study that might represent the common set of* MYC*-regulated genes in gastric carcinogenesis (Table 4). This is important because it shows that, even though we have represented distinct GC histological subtypes and disease stages, there is still a core set of genes regulated by MYC involved in the carcinogenic process. We also did not find other reports in the literature concerning those 14 genes and high-throughput analysis of gastric cancer [1, 22, 26, 44–46, 53, 59–64]. Even when we consider the molecular signatures presented by Brazilian intestinal-type GC [47], we could not find any concordance for the 14 genes found by our study, but it is important to consider that Binato et al. [47] did not take into account* MYC* overexpression in their samples. Therefore, the unique DEGs found in this paper represent new and important findings concerning the process of gastric carcinogenesis regulated by MYC in the Brazilian population.

Even though additional studies are needed to validate our results, we present strong evidence that MYC-regulated genes in GC have different expression patterns when we consider histological and disease stage differences; however, they still share pathways and core genes involved in the carcinogenic process.

## Figures and Tables

**Table 1:** Samples used in this study. Control siRNA samples are labeled as C and MYC-siRNA as M.

Cell line	Samples (GEO)	Sample Name	GC Histological subtype
AGP01	GSM2147866	1C	Ascitic fluid of intestinal GC
	GSM2147867	1M	Ascitic fluid of intestinal GC
ACP02	GSM2147868	2C	Diffuse
	GSM2147869	2M	Diffuse
ACP03	GSM2147870	3C	Intestinal
	GSM2147871	3M	Intestinal

**Table 2:** Reads quantification after *MYC*-siRNA.

Sample name	Total Reads	Total Mapped Reads	Gene mapping rate	Expressed genes
1C	13.319.736	12.944.452	97.18%	14.594
1M	11.043.607	10.771.298	97.53%	10.709
2C	12.021.142	11.751.830	97.76%	9.859
2M	11.701.695	11.481.863	98.12%	8.793
3C	12.772.535	12.546.479	98.23%	7.988
3M	12.430.807	12.112.157	97.44%	10.685

**Table 3:** Enriched hallmark gene sets for DEGs after *MYC*-siRNa in three GC cell lines.

Hallmark Name:	Process category	Description	Size^∗^	ES	NES	Nom. P-value	FDR	Max. Rank
MYC_TARGETS_V1	Proliferation	MYC targets, variant 1	100/44	0.59	1.89	0.00	0.000	1368
REACTIVE_OXIGEN_SPECIES_PATHWAY	Pathway	Reactive oxygen species pathway	25/13	0.55	1.42	0.00	0.000	2316
UNFOLDED_PROTEIN_RESPONSE	Pathway	Unfolded protein response; ER stress	50/22	0.55	1.56	0.00	0.000	1891
PROTEIN_SECRETION	Pathway	Protein secretion	44/26	0.55	1.70	0.00	0.000	2376
G2M_CHECKPOINT	Proliferation	Cell cycle progression: G2/M checkpoint	97/40	0.52	1.67	0.00	0.000	1813
MTORC1_SIGNALING	Signaling	mTORC1 signaling	102/43	0.50	1.46	0.00	0.044	2021
E2F_TARGETS	Proliferation	Cell cycle progression: E2F targets	89/35	0.48	1.42	0.00	0.052	1607
OXIDATIVE_PHOSPHORYLATION	Metabolic	Oxidative phosphorylation and citric acid cycle	118/57	0.48	1.46	0.00	0.062	2284
CHOLESTEROL_HOMEOSTASIS	Metabolic	Cholesterol homeostasis	39/14	0.48	1.48	0.00	0.069	1671
DNA_REPAIR	DNA damage	DNA repair	80/38	0.46	1.42	0.00	0.077	2337
APOPTOSIS	Pathway	Programmed cell death; caspase pathway	66/29	0.42	1.31	0.20	0.190	2405

^*∗*^Number of genes found for each gene set after curating data. ES: enrichment score. NES: normalized enrichment score. Nom. P-value: nominal P-values for each gene set. FDR: false discovery rate. Max. rank: maximum rank numbers for each gene sets.

**Table 4:** The 14 common DEGs for three GC cell lines after *MYC*-siRNA and the gene set hallmarks they were enriched in.

**Hallmark Name:**	**Gene Symbol**	**Rank in Gene List**	**Rank Metric Score**	**Running (ES)**
PROTEIN_SECRETION	*BNIP3*	1438	6.0E-01	0.522
UNFOLDED_PROTEIN_RESPONSE	*SKIV2L2*	1051	7.2E-01	0.508
UNFOLDED_PROTEIN_RESPONSE	*SRPRB*	1442	6.0E-01	0.506
REACTIVE_OXIGEN_SPECIES_PATHWAY	*JUNB*	1969	4.8E-01	0.491
G2M_CHECKPOINT	*LBR*	817	8.2E-01	0.451
OXIDATIVE_PHOSPHORYLATION	*NDUFV2*	1542	5.7E-01	0.444
APOPTOSIS	*CDKN1B*	2405	4.0E-01	0.420
PROTEIN_SECRETION	*RAB22A*	289	1.2E+15	0.408
CHOLESTEROL_HOMEOSTASIS	*ATXN2*	945	7.6E-01	0.382
CHOLESTEROL_HOMEOSTASIS	*LGMN*	445	1.1E+16	0.367
OXIDATIVE_PHOSPHORYLATION	*ACAT1*	840	8.1E-01	0.367
PROTEIN_SECRETION	*TMED2*	254	1.3E+16	0.326
DNA_REPAIR	*AAAS*	1109	7.0E-01	0.313
G2M_CHECKPOINT	*NCL*	328	1.2E+13	0.289

ES: enrichment score.

## Data Availability

The gene expression data used to support the findings of this study have been deposited in the Gene Expression Omnibus (GEO) repository under the access number GSE81265 (https://www.ncbi.nlm.nih.gov/geo/query/acc.cgi?acc=GSE81265). A detailed description of each gene set can be found within the paper at Table 1.

## Supplementary Materials

Supplementary Materials Table 1: enriched hallmark gene sets for DEGs. Table 2: enriched KEGG gene sets for DEGs. Table 3: the 14 common DEGs for three GC cell lines after* MYC* siRNA and the gene set hallmarks they were enriched and identified in cancer-associated studies.
