# Supplementary material for: Gastric Cancer Cell Lines Have Different MYC-Regulated Expression Patterns but Share a Common Core of Altered Genes
Source: Can J Gastroenterol Hepatol. 2018 Oct 16;2018:5804376. doi: 10.1155/2018/5804376 (PMC6206580; doi:10.1155/2018/5804376)
Supplement: Supplementary Materials — Table 1: enriched hallmark gene sets for DEGs. Table 2: enriched KEGG gene sets for DEGs. Table 3: the 14 common DEGs for three GC cell lines after MYC siRNA and the gene set hallmarks they were enriched and identified in cancer-associated studies. [file 5804376.f1.docx]

**Supplementary Materials**

**Table 1:** Enriched Hallmark gene sets for DEGs.

| **ACP02: Diffuse** | | | | |
| --- | --- | --- | --- | --- |
| **Gene set** | **Gene Symbol** | **Rank in Gene List** | **Rank Metric Score** | **Running (ES)** |
| MYC_TARGETS_V1 | *SNRPD2* | 1.368 | 6.2E-01 | 0.59 |
| REACTIVE_OXIGEN_SPECIES_PATHWAY | *GPX3* | 2.316 | 4.2E-01 | 0.55 |
| UNFOLDED_PROTEIN_RESPONSE | *NFYB* | 1.891 | 4.9E-01 | 0.55 |
| PROTEIN_SECRETION | *RER1* | 2.334 | 4.2E-01 | 0.54 |
| G2M_CHECKPOINT | *RBM14* | 1.686 | 5.4E-01 | 0.52 |
| MTORC1_SIGNALING | *YKT6* | 2.021 | 4.7E-01 | 0.50 |
| CHOLESTEROL_HOMEOSTASIS | *EBP* | 1.671 | 5.4E-01 | 0.48 |
| OXIDATIVE_PHOSPHORYLATION | *ATP5F1* | 2.181 | 4.4E-01 | 0.47 |
| E2F_TARGETS | *DEPDC1* | 1.582 | 5.6E-01 | 0.47 |
| DNA_REPAIR | *NME3* | 2.311 | 4.2E-01 | 0.45 |
| APOPTOSIS | *CDKN1B* | 2.405 | 4.0E-01 | 0.42 |
|  | | | | |
| **ACP03: Intestinal** | | | | |
| **Gene set** | **Gene Symbol** | **Rank in Gene List** | **Rank Metric Score** | **Running (ES)** |
| MYC_TARGETS_V1 | *TYMS* | 1.365 | 6.2E-01 | 0.59 |
| PROTEIN_SECRETION | *KIF1B* | 2.376 | 4.1E-01 | 0.55 |
| REACTIVE_OXIGEN_SPECIES_PATHWAY | *GPX3* | 2.316 | 4.2E-01 | 0.55 |
| UNFOLDED_PROTEIN_RESPONSE | *EDEM1* | 1.752 | 5.3E-01 | 0.53 |
| G2M_CHECKPOINT | *DBF4* | 1.813 | 5.1E-01 | 0.52 |
| MTORC1_SIGNALING | *YKT6* | 2.021 | 4.7E-01 | 0.50 |
| CHOLESTEROL_HOMEOSTASIS | *EBP* | 1.671 | 5.4E-01 | 0.48 |
| E2F_TARGETS | *TIMELESS* | 1.568 | 5.7E-01 | 0.46 |
| DNA_REPAIR | *RNMT* | 2.194 | 4.4E-01 | 0.45 |
| OXIDATIVE_PHOSPHORYLATION | *NDUFS8* | 2.038 | 4.7E-01 | 0.45 |
| APOPTOSIS | *CDKN1B* | 2.405 | 4.0E-01 | 0.42 |
|  | | | | |
| **AGP01: Ascitic fluid of an intestinal (Metastatic)** | | | | |
| **Gene set** | **Gene Symbol** | **Rank in Gene List** | **Rank Metric Score** | **Running (ES)** |
| MYC_TARGETS_V1 | *TYMS* | 1.365 | 6.2E-01 | 0.59 |
| PROTEIN_SECRETION | *KIF1B* | 2.376 | 4.1E-01 | 0.55 |
| REACTIVE_OXIGEN_SPECIES_PATHWAY | *GPX3* | 2.316 | 4.2E-01 | 0.55 |
| UNFOLDED_PROTEIN_RESPONSE | *NFYB* | 1.891 | 4.9E-01 | 0.55 |
| G2M_CHECKPOINT | *RBM14* | 1.686 | 5.4E-01 | 0.52 |
| MTORC1_SIGNALING | *YKT6* | 2.021 | 4.7E-01 | 0.50 |
| OXIDATIVE_PHOSPHORYLATION | *ACADSB* | 2.284 | 4.3E-01 | 0.48 |
| CHOLESTEROL_HOMEOSTASIS | *EBP* | 1.671 | 5.4E-01 | 0.48 |
| E2F_TARGETS | *TIMELESS* | 1.568 | 5.7E-01 | 0.46 |
| DNA_REPAIR | *ERCC3* | 2.142 | 4.5E-01 | 0.44 |
| APOPTOSIS | *CDKN1B* | 2.405 | 4.0E-01 | 0.42 |

ES: enrichment score.

**Table 2:** Enriched KEGG gene sets for DEGs.

| **ACP02: Diffuse** | | | | |
| --- | --- | --- | --- | --- |
| **KEGG Gene sets** | **Gene Symbol** | **Rank in Gene List** | **Rank Metric Score** | **Running (ES)** |
| RIBOSOME | *RPS15* | 1.233 | 7.1E-01 | 0.69 |
| DORSO_VENTRAL_AXIS_FORMATION | *SOS2* | 996 | 6.0E-01 | 0.62 |
| DNA_REPLICATION | *RNASEH2B* | 2.165 | 4.5E-01 | 0.61 |
| STARCH_AND_SUCROSE_METABOLISM | *PYGL* | 1.386 | 6.1E-01 | 0.61 |
| NOTCH_SIGNALING_PATHWAY | *DVL3* | 837 | 8.1E-01 | 0.61 |
| OXIDATIVE_PHOSPHORYLATION | *ATP6V0A2* | 2.289 | 4.3E-01 | 0.57 |
| AMINO_SUGAR_AND_NUCLEOTIDE_SUGAR_METABOLISM | *AMDHD2* | 521 | 1.0E+16 | 0.55 |
| EPITHELIAL_CELL_SIGNALING_IN_HELICOBACTER_PYLORI_INFECTION | *ATP6V0A2* | 2.289 | 4.3E-01 | 0.50 |
| CELL_CYCLE | *CDK4* | 2.549 | 3.8E-01 | 0.50 |
| SPLICEOSOME | *PRPF18* | 1.550 | 5.7E-01 | 0.49 |
| INTESTINAL_IMMUNE_NETWORK_FOR_IGA_PRODUCTION | *MAP3K14* | 1.984 | 4.8E-01 | 0.46 |
| UBIQUITIN_MEDIATED_PROTEOLYSIS | *UBE2J2* | 2.568 | 3.7E-01 | 0.44 |
|  | | | | |
| **ACP03: Intestinal** | | | | |
| **KEGG Gene sets** | **Gene Symbol** | **Rank in Gene List** | **Rank Metric Score** | **Running (ES)** |
| RIBOSOME | *RPL12* | 1.159 | 6.8E-01 | 0.68 |
| DORSO_VENTRAL_AXIS_FORMATION | *SOS1* | 1.415 | 6.0E-01 | 0.65 |
| STARCH_AND_SUCROSE_METABOLISM | *PYGL* | 1.386 | 6.1E-01 | 0.61 |
| DNA_REPLICATION | *RNASEH2C* | 1.809 | 5.1E-01 | 0.60 |
| NOTCH_SIGNALING_PATHWAY | *NOTCH2* | 654 | 9.0E-01 | 0.57 |
| OXIDATIVE_PHOSPHORYLATION | *ATP6V0A2* | 2.289 | 4.3E-01 | 0.57 |
| SPLICEOSOME | *DHX16* | 1.569 | 5.7E-01 | 0.50 |
| EPITHELIAL_CELL_SIGNALING_IN_HELICOBACTER_PYLORI_INFECTION | *ATP6V0A2* | 2.289 | 4.3E-01 | 0.50 |
| CELL_CYCLE | *ANAPC1* | 2.493 | 3.9E-01 | 0.50 |
| INTESTINAL_IMMUNE_NETWORK_FOR_IGA_PRODUCTION | *MAP3K14* | 1.984 | 4.8E-01 | 0.46 |
| UBIQUITIN_MEDIATED_PROTEOLYSIS | *UBE2J2* | 2.568 | 3.7E-01 | 0.44 |
| AMINO_SUGAR_AND_NUCLEOTIDE_SUGAR_METABOLISM | *GPI* | 354 | 1.2E+16 | 0.42 |
|  | | | | |
| **AGP01: Ascitic fluid of an intestinal (Metastatic)** | | | | |
| **KEGG Gene sets** | **Gene Symbol** | **Rank in Gene List** | **Rank Metric Score** | **Running (ES)** |
| RIBOSOME | *RPS11* | 1.086 | 7.1E-01 | 0.67 |
| DORSO_VENTRAL_AXIS_FORMATION | *SOS1* | 1.415 | 7.4E-01 | 0.65 |
| AMINO_SUGAR_AND_NUCLEOTIDE_SUGAR_METABOLISM | *GNPDA1* | 831 | 8.1E-01 | 0.63 |
| STARCH_AND_SUCROSE_METABOLISM | *PYGL* | 1.386 | 6.1E-01 | 0.61 |
| NOTCH_SIGNALING_PATHWAY | *DVL3* | 837 | 8.1E-01 | 0.61 |
| DNA_REPLICATION | *RNASEH2C* | 1.809 | 5.1E-01 | 0.60 |
| OXIDATIVE_PHOSPHORYLATION | *ATP6V0A2* | 2.289 | 4.3E-01 | 0.57 |
| INTESTINAL_IMMUNE_NETWORK_FOR_IGA_PRODUCTION | *IL6* | 2.064 | 4.7E-01 | 0.52 |
| EPITHELIAL_CELL_SIGNALING_IN_HELICOBACTER_PYLORI_INFECTION | *ATP6V0A2* | 2.289 | 4.4E-01 | 0.50 |
| CELL_CYCLE | *CDK4* | 2.549 | 3.8E-01 | 0.50 |
| SPLICEOSOME | *SNRPF* | 1.167 | 6.8E-01 | 0.46 |
| UBIQUITIN_MEDIATED_PROTEOLYSIS | *ANAPC1* | 2.493 | 3.9E-01 | 0.42 |

ES: enrichment score.

**Table 3:** The 14 common DEGs for three GC cell lines after MYC-siRNA and the gene set hallmarks they were enriched and identified in cancer-associated studies.

| **Hallmark Name:** | **Gene Symbol** | **Rank in Gene List** | **Rank Metric Score** | **Running (ES)** |
| --- | --- | --- | --- | --- |
| PROTEIN_SECRETION | *BNIP3* [1] | 1438 | 6.0E-01 | 0.522 |
| UNFOLDED_PROTEIN_RESPONSE | *SKIV2L2* [2] | 1051 | 7.2E-01 | 0.508 |
| UNFOLDED_PROTEIN_RESPONSE | *SRPRB* [3] | 1442 | 6.0E-01 | 0.506 |
| REACTIVE_OXIGEN_SPECIES_PATHWAY | *JUNB* [4] | 1969 | 4.8E-01 | 0.491 |
| G2M_CHECKPOINT | *LBR* [5] | 817 | 8.2E-01 | 0.451 |
| OXIDATIVE_PHOSPHORYLATION | *NDUFV2* [6] | 1542 | 5.7E-01 | 0.444 |
| APOPTOSIS | *CDKN1B* [7] | 2405 | 4.0E-01 | 0.420 |
| PROTEIN_SECRETION | *RAB22A* [8] | 289 | 1.2E+15 | 0.408 |
| CHOLESTEROL_HOMEOSTASIS | *ATXN2* [9] | 945 | 7.6E-01 | 0.382 |
| CHOLESTEROL_HOMEOSTASIS | *LGMN* [10] | 445 | 1.1E+16 | 0.367 |
| OXIDATIVE_PHOSPHORYLATION | *ACAT1*[11] | 840 | 8.1E-01 | 0.367 |
| PROTEIN_SECRETION | *TMED2* [12] | 254 | 1.3E+16 | 0.326 |
| DNA_REPAIR | *AAAS* [13] | 1109 | 7.0E-01 | 0.313 |
| G2M_CHECKPOINT | *NCL* [14] | 328 | 1.2E+13 | 0.289 |

ES: enrichment score.

**References**

1. Erkan M, Kleeff J, Esposito I, Giese T, Ketterer K, Büchler MW, et al. Loss of BNIP3 expression is a late event in pancreatic cancer contributing to chemoresistance and worsened prognosis. Oncogene. 2005;24: 4421–4432. doi:10.1038/sj.onc.1208642

2. Onderak AM, Anderson JT. Loss of the RNA helicase SKIV2L2 impairs mitotic progression and replication-dependent histone mRNA turnover in murine cell lines. RNA. 2017;23: 910–926. doi:10.1261/rna.060640.117

3. Sehgal P, Kumar N, Rajesh V, Kumar P, Patil S, Bhattacharya A, et al. Regulation of protumorigenic pathways by Insulin like growth factor binding protein2 and its association along with β -catenin in breast cancer lymph node metastasis. 2013; 1–14.

4. Thomsen MK, Bakiri L, Hasenfuss SC, Wu H, Morente M, Wagner EF. Loss of JUNB/AP-1 promotes invasive prostate cancer. Cell Death Differ. 2015;22: 574–582. doi:10.1038/cdd.2014.213

5. Sakthivel KM, Sehgal P. A novel role of lamins from genetic disease to cancer biomarkers. Oncol Rev. 2016;10: 65–71. doi:10.4081/oncol.2016.309

6. Su CY, Chang YC, Yang CJ, Huang MS, Hsiao M. The opposite prognostic effect of NDUFS1 and NDUFS8 in lung cancer reflects the oncojanus role of mitochondrial complex i. Sci Rep. Nature Publishing Group; 2016;6: 6–13. doi:10.1038/srep31357

7. Canbay E, Eraltan IY, Cercel A, Isbir T, Gazioglu E, Aydogan F, et al. CCND1 and CDKN1B polymorphisms and risk of breast cancer. Anticancer Res. 2010;30: 3093–3098.

8. Wang T, Gilkes DM, Takano N, Xiang L, Luo W, Bishop CJ, et al. Hypoxia-inducible factors and RAB22A mediate formation of microvesicles that stimulate breast cancer invasion and metastasis. Proc Natl Acad Sci. 2014;111: E3234–E3242. doi:10.1073/pnas.1410041111

9. Drost J, Nonis D, Eich F, Leske O, Damrath E, Brunt ER, et al. Ataxin-2 modulates the levels of Grb2 and Src but not ras signaling. J Mol Neurosci. 2013;51: 68–81. doi:10.1007/s12031-012-9949-4

10. Zhen Y, Chunlei G, Wenzhi S, Shuangtao Z, Na L, Rongrong W, et al. Clinicopathologic significance of legumain overexpression in cancer: A systematic review and meta-analysis. Sci Rep. Nature Publishing Group; 2015;5: 1–9. doi:10.1038/srep16599

11. Garcia-Bermudez J, Birsoy K. Drugging ACAT1 for Cancer Therapy. Mol Cell. 2016;64: 856–857. doi:10.1016/j.molcel.2016.11.023

12. Shi-peng G, Chun-lin C, Huan W, Fan-liang M, Yong-ning C, Ya-di Z, et al. TMED2 promotes epithelial ovarian cancer growth. Oncotarget. 2017;8: 94151–94165. doi:10.18632/oncotarget.21593

13. Brown B, Agdere L, Muntean C, David K. Alacrima as a harbinger of adrenal insufficiency in a child with allgrove (AAA) syndrome. Am J Case Rep. 2016;17: 703–706. doi:10.12659/AJCR.899546

14. Pichiorri F, Palmieri D, De Luca L, Consiglio J, You J, Rocci A, et al. In vivo NCL targeting affects breast cancer aggressiveness through miRNA regulation. J Exp Med. 2013;210: 951–968. doi:10.1084/jem.20120950
